# Shortening‐induced force depression is modulated in a time‐ and speed‐dependent manner following a stretch–shortening cycle

**DOI:** 10.14814/phy2.13279

**Published:** 2017-06-30

**Authors:** Rafael Fortuna, Martin Groeber, Wolfgang Seiberl, Geoffrey A. Power, Walter Herzog

**Affiliations:** ^1^ Human Performance LaboratoryFaculty of KinesiologyUniversity of CalgaryCalgaryCanada; ^2^ Department of Biomechanics in SportsFaculty of Sport and Health SciencesTechnische Universität MünchenMunichGermany; ^3^ Neuromechanical Performance Research LabDepartment of Human Health and Nutritional SciencesCollege of Biological SciencesUniversity of GuelphGuelphOntarioCanada

**Keywords:** Concentric, cross‐bridge theory, eccentric, electrical stimulation, force depression, mechanisms of contraction, residual force enhancement

## Abstract

The steady‐state isometric force following active muscle shortening or lengthening is smaller (force depression, FD) or greater (residual force enhancement, RFE) than a purely isometric contraction at the corresponding length. The mechanism underlying these phenomena is not explained within the context of the cross‐bridge theory, with few studies investigating the effects of FD and RFE in stretching–shortening cycle (SSC). The purpose of this study was to perform SSC, where the time between the end of stretch and the end of shortening was manipulated by (1) adding a pause between stretch and shortening (protocol 1) or (2) performing the shortening contraction at different speeds (protocol 2). The results show that, in protocol 1, FD was reduced for SSC with a 0‐sec and 0.5‐sec interval between stretching and shortening, but was the same for SSC with a 1‐sec interval compared to the pure FD condition. In protocol 2, FD was reduced for SSC with shortening speeds of 30 and 60°/sec, but was the same for shortening speeds of 15 and 20°/sec compared to the pure FD condition. These findings provide evidence that stretch preceding shortening affects FD in a time‐ and speed*‐*dependent manner, providing new information on the potential mechanism of FD and RFE.

## Introduction

Muscle force production has been shown to be history dependent (Abbott and Aubert 1952; Maréchal and Plaghki 1979; Edman et al. 1982; Joumaa et al. 2008; Herzog, 2014). History‐dependent muscle properties cause that the steady‐state isometric force following an active shortening or lengthening contraction is smaller (force depression; FD) or greater (residual force enhancement; RFE) compared to a purely isometric contraction at the corresponding muscle length and same level of activation (Abbott and Aubert 1952; Maréchal and Plaghki 1979; Edman et al. 1982). Despite being well‐documented across all structural levels of muscle over the last 50 years, and observed during electrically stimulated (Lee et al. 2001; Seiberl et al. 2015; Fortuna et al. 2016) and voluntary contractions in humans (Lee and Herzog 2002; Power et al. 2014a,b), the mechanism behind these history‐dependent phenomena remains unknown.

Residual force enhancement (RFE) has been shown to depend on stretch magnitude but not stretch speed, is long‐lasting (Abbott and Aubert 1952; Maréchal and Plaghki 1979; Edman et al. 1982; Pinniger et al. 2006; Cornachione and Rassier 2012), and has been suggested to be associated with the engagement of a passive element upon muscle activation, thereby increasing muscle stiffness (Herzog and Leonard 2002; Labeit et al. 2003; Joumaa et al. 2008; DuVall et al. 2013). Force depression (FD) has been shown to depend on the magnitude, speed, and force of shortening, it is long‐lasting but can be abolished instantaneously if activation is interrupted for long enough for force to drop to zero, and it is proportional to the amount of work performed (Herzog and Leonard 1997; Herzog et al. 1998). Maréchal and Plaghki (1979) proposed that a deformation in the compliant actin filaments entering the newly formed overlap zone during the shortening phase causes a change in the relative orientation between myosin cross‐bridges and actin‐binding sites, thereby reducing the probability for cross‐bridge attachments in the newly formed overlap zone (Maréchal and Plaghki 1979). This mechanism is supported by the observation that stiffness is decreased proportionally with the magnitude of FD, resulting in fewer attached cross‐bridges in the FD state as compared with a purely isometric reference contraction (Leonard et al. 2010).

Investigations into the mechanisms of RFE and FD are usually performed separately, with few studies combining stretching and shortening or vice versa (Herzog and Leonard 2000; Rassier and Herzog 2004; Seiberl et al. 2015). During activities of daily living, muscles undergo shortening–stretching and stretching–shortening contractions on a regular basis. Therefore, determining how stretching preceding shortening affects force production might help us to better understand the functional implications of history‐dependent properties, and might also provide novel insights into the mechanisms underlying the history dependence of muscle contraction. Previous studies showed that shortening–stretching and stretching–shortening cycles in the cat soleus muscle are not commutative with respect to the isometric force (Herzog and Leonard 2000; Lee et al. 2001). Herzog and Leonard (2000) reported that RFE following stretch depends on the amount of muscle shortening preceding the stretch, whereas FD following shortening appeared to be unaffected by previous stretching of the muscle. The results for shortening–stretching cycles seem to fit nicely into simple rheological models of muscle contraction (Forcinito et al. 1998), which account for history‐dependent muscle properties through the engagement of a passive structural element upon activation. However, the same model could not explain the results observed for stretching–shortening cycles in the cat soleus, with the authors suggesting that the results may hold an important clue for the mechanism associated with RFE and FD.

Contrary to the results reported by Herzog and Leonard (2000) and Lee et al. (2001), Seiberl et al. (2015) found that RFE was abolished during the shortening phase of stretch–shortening cycles (SSCs) for fast stretch–shortening contractions, with the authors suggesting that the RFE acquired during active muscle stretching might have been abolished in a transient manner during shortening. However, it was not clear if the transient disappearance of RFE depends on the shortening speed or is merely a function of time. Therefore, the purpose of the present study was to revisit the history‐dependent properties of SSC contractions in electrically stimulated human adductor pollicis muscles, and to test if either the time between SSC and/or the speed of shortening play a role in the steady‐state force postshortening.

## Methods

### Participants

Sixteen healthy subjects (8 males and 8 females; age 25 ± 2 years; height 170 ± 9 cm; weight 67 ± 8 kg) participated in protocol 1, while protocol 2 had 15 subjects with similar group characteristics (8 males and 7 females; age 26 ± 4 years; height 173 ± 8 cm; weight 60 ± 10 kg). Subjects had no history of neuromuscular disorders or hand injuries and gave free, written informed consent to participate in this study. All experimental procedures were approved by the Conjoint Ethics Committee of the University of Calgary and conformed to the Declaration of Helsinki. The participants were familiar with the procedures and techniques used.

### Experimental setup

Thumb adduction forces and carpometacarpal angular displacements were measured using a custom‐designed dynamometer (Lee and Herzog 2002; Fortuna et al. 2012; Jones et al. 2016). The left hand was immobilized with a reusable clinical cast (Ezeform, Rehabilitation Division, Smith & Nephew, Inc., Germantown, WI) and was secured with two inelastic straps, restricting movement of the wrist and finger except of the thumb. Participants sat on an adjustable chair with the shoulder slightly abducted and the elbow flexed at 90°. A rotary stepper motor (Model TS42BP10 Parker Hannifin Corp., Cleveland, OH) was connected to an aluminum rod (1.5 cm diameter and 15 cm long), via gears (1:4 gears ratio). The other end of the rod was attached to an auxiliary piece for thumb placement and fixation. During the experiment, the thumb pressed against the auxiliary piece which was in line with the direction of force measurement obtained through two pairs of calibrated strain gauges (Model CEA‐06–125UN‐350, Measurement Group, Inc., Raleigh, NC). The forearm was slightly supinated relative to the thumb placed on the auxiliary piece, thus thumb movement was guided toward the third finger. A 0° reference angle was defined for each subject as the highest degree of thumb adduction possible before the dynamometer arm came in contact with the cast. Thumb angles increased with abduction, up to 30°, which was the maximum abduction angle that could be reached comfortably by all subjects.

### Electrical stimulation

Two self‐adhering Ag‐AgCl surface electrodes (2 × 3 cm) were placed over the ulnar nerve to electrically activate the adductor pollicis muscle, with the cathode 2 cm proximal to the bone on the medial wrist and the anode 2 cm proximal to the cathode. A Grass S8000 stimulator (Astro Med, Inc., Longueil, Quebec, Canada) was used to increase intensity until no further increase in twitch force was observed (single 100 *μ*sec square‐wave pulses). Next, a maximum voluntary contraction (MVC) at 0° was obtained for 3 sec. All subsequent contractions were electrically evoked. Tetanic electrical stimulation for all contractions was performed with an identical setup. The voltage of nerve stimulation was increased (fixed frequency: 50 Hz) until force reached 50–60% of the participant's MVC, and was kept constant throughout the experimental period. The adjusted voltage intensity was used for 7 sec stimulations during each evoked contraction.

### Experimental protocols 1 and 2

The protocols consisted of a force depression (FD) test, followed by separate and randomized SSCs. Each protocol finished with a final MVC to assess potential effects of fatigue.

Force depression (FD) test: the muscle was preactivated and held isometrically for 1 sec at 30°, followed by 1 sec of shortening over a 30° joint excursion at an angular velocity of 30°/sec. The muscle was then held isometrically at 0° for 5 sec. Following the FD test, a purely isometric reference contraction was performed at 0° for 7 sec.

### Protocol 1

**Figure 1:**
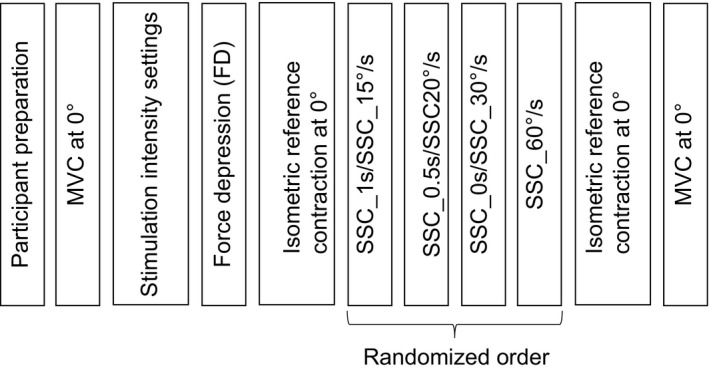
Experimental design for protocols 1 and 2. Following participant preparation, maximum voluntary contraction (MVC) was performed at 0° thumb adduction angle. Stimulation intensity was set to 50–60% of peak MVC force. Protocol 1 and 2 started with force depression followed by the appropriate isometric test. Next, a randomized order of stretch‐shortening cycles (SSC)s with either a (i) varying time interval between stretching and shortening (SSC_0s, SSC_0.5s, SSC_1s; protocol 1) or (ii) varying stretch speeds (SSC_15°/s, SSC_20°/s, SSC_30°/s, SSC_60°/s; protocol 2). Last, an isometric reference contraction at 0° and a MVC were performed to assess fatigue.

SSC test 1: the muscle was preactivated and held isometric for 1 sec at 0° and was stretched to 30° at 30°/sec. Following stretching, the muscle was then either immediately shortened back to 0° at 30°/sec (SSC_0s), held isometrically for 0.5 sec (SSC_0.5s) then shortened back to 0°, or held isometrically for 1 sec (SSC_1s) then shortened back to 0°. Following shortening, all SSCs (SSC_0s, SSC_0.5s, and SSC_1s) were held isometrically at 0° angle for 4, 3.5, and 3 sec, respectively. The order of each trial condition was randomized (Fig 1).

### Protocol 2

SSC test 2: the muscle was preactivated and held isometric for 1 sec at 0° and was stretched to 30° at 30°/sec. Following stretching, the muscle was then shortened back immediately to 0° at either 15°/sec (SSC_15°/s), 20°/sec (SSC_20°/s), 30°/sec (SSC_30°/s), or 60°/s (SSC_60°/s), and was held isometrically at the 0° angle for 3, 3.5, 4, and 4.5 sec, respectively. The order of each trial was randomized (Fig 1).

Following the SSCs of protocols 1 and 2, an isometric reference contraction lasting 7 sec and an MVC at a thumb adduction angle of 0° was performed to ensure there was no muscle fatigue. A 5‐min rest interval was provided between trials.

### Data reduction and analysis

**Figure 2:**
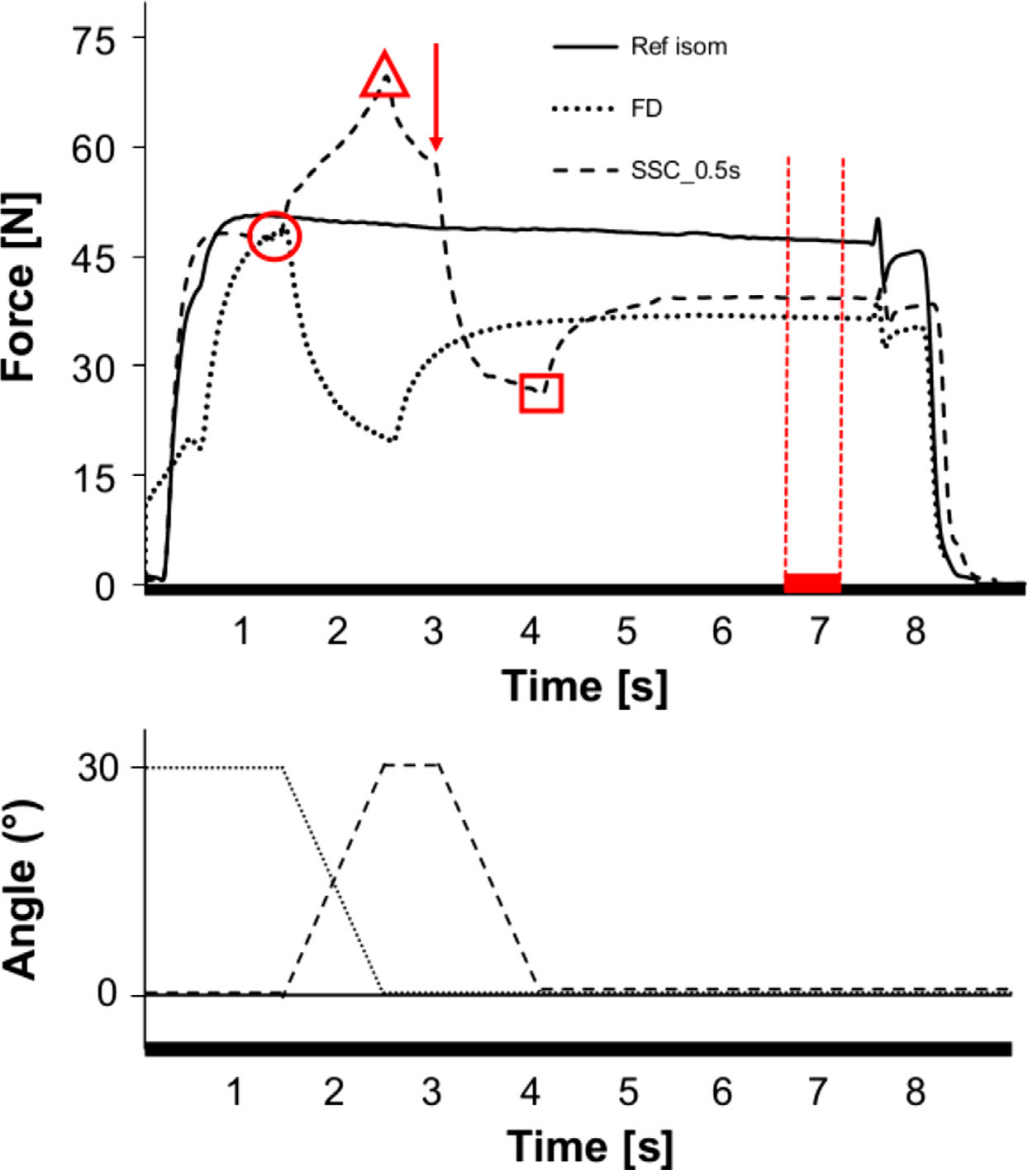
Thumb adduction force (top) and metacarpophalangeal joint angle (bottom) as a function of time for an exmplar isometric reference contraction at 0° thumb abduction (solid line, Ref isom), a pure shortening‐induced force depression (dotted line, FD) and a stretch‐shortening cycle (SSC) in which shortening follows the stretching phase after 500msec delay (↓ start of shortening) at a 30°/s stretch velocity (dashed line, SSC_0.5s). The average force (mean 200msec) before stretching (b‐STR, •), the maximum force at the end of the stretch (e‐STR;▲), the minimum force at end of shortening (e‐SHO; ■) and the average isometric steady‐state force (red vertical dotted line; 500msec) prior to muscle deactivation were assessed.

During testing, thumb adduction force and thumb joint angle were collected at a sampling frequency of 2000 Hz per channel, and saved on a computer with an analog to digital converter. Force data were filtered (low pass 10 Hz). Force prior to stretching (200 msec average; b‐STR), at the end of the stretch (maximum force; e‐STR), at the end of shortening (minimum force; e‐SHO), and the mean force during a 500‐msec period prior to muscle deactivation were used for statistical analysis (Fig. 2). The mean force values for the SSCs were compared to the corresponding values obtained from the isometric reference contraction and to the pure shortening (FD) without prior stretching. Additionally, work during the shortening phase for pure shortening (FD) and the SSCs was calculated as the product of mean force during shortening, the length of the lever arm, and the joint angular displacement (30°).

Data were tested for normality (Shapiro–Wilk test) and a one‐way ANOVA with post hoc correction (Holm‐Sidak test) was used to identify significant differences between parameters of (mean) forces, work during shortening, comparisons of force obtained in the SSCs, and the purely isometric reference contractions. Statistical significance was set at *α *= 0.05.

## Results

### Protocol 1

The mean electrically evoked tetanic isometric force at a 0° thumb adduction angle was 38.9 ± 7.5 N, representing 52.5% of the MVC (74.1 ± 14.1 N). There was a 5% loss in isometric force from before to after the SSC contractions.

As expected, owing to the starting length of contractions, there was a significantly greater force before stretching (b‐STR) for SSC contraction compared to the initial force before shortening for pure FD contractions (*P *˂ 0.05; Fig 2), but no difference in force among SSC contractions (*P *>* *0.05). Additionally, SSC contractions reached a similar value of peak force at the end of the stretch (e‐STR) (*P *>* *0.05). Force at the end of shortening (e‐SHO) was significantly higher for SSC compared to FD contractions, with SSC_0s showing greater forces than SSC_1s (*P* ˂ 0.05). Finally, there was significantly more work performed for all SSC compared to the FD contractions (*P* ˂ 0.05), with the most work performed for the SSC_0s (Table 1).

**Table 1:** Mean (±SE) values of work, force before stretching (b‐STR), end of stretching (e‐STR), and end of shortening (e‐SHO) for FD, SSC_0s, SSC_0.5s, and SSC_1s for protocol 1

Contraction condition	Work [J]		Force b‐STR [N]		Force e‐STR [N]		Force e‐SHO [N]	
	Mean	SE	Mean	SE	Mean	SE	Mean	SE
FD	2.68	0.8	44.8	3.0	—	—	19.6	1.9
SSC_0s	3.59^a^^,^ b	0.7	40.6	2.1	66.1	3.6	24.5^a^^,^ c	2.1
SSC_0.5s	3.27^a^^,^ b	0.8	40.6	2.0	64.0	3.3	23.9^a^	1.8
SSC_1s	2.93^a^	0.5	40.9	1.9	65.0	3.5	23.1^a^	1.9

a Compared to force depression (FD).

b Compared to SCC_1 s.

c Compared to SSC_1 s.

**Table 2:** Mean (±SE) values of work, force before stretching (b‐STR), end of stretching (e‐STR), and end of shortening (e‐SHO) for force depression (FD), SSC_0s, SSC_0.5s, and SSC_1s for protocol 2

Contraction condition	Work [J]		Force b‐STR [N]		Force e‐STR [N]		Force e‐SHO [N]	
	Mean	SE	Mean	SE	Mean	SE	Mean	SE
FD	1.52	0.4	47.0	3.0	—	—	19.7	2.0
SSC_15°/s	2.05^a^^,^ b	0.4	40.1^a^	2.8	65.9	3.0	26.1^a^^,^ b	2.0
SSC_20°/s	1.95^a^	0.4	39.4^a^	2.6	63.7	3.6	25.0^a^^,^ b	1.9
SSC_30°/s	1.96^a^	0.4	40.5^a^	2.8	65.3	3.6	25.0^a^^,^ b	2.0
SSC_60°/s	1.75^a^	0.3	38.4^a^	2.3	63.9	3.2	19.6	1.4

a Compared to force depression (FD).

b Compared to SCC_60°/s.

**Figure 3:**
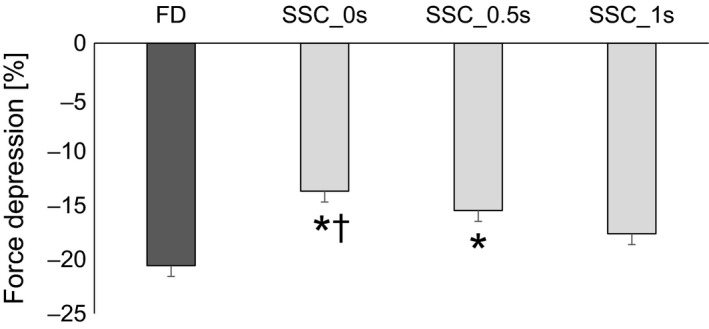
Mean (±SE) values of force decrease for pure force depression (FD) and stretching‐shortening cycles (SSC) with 0, 0.5, and 1s interval between stretching and shortening (SSC_0s, SSC_0.5s, SSC_1s, respectively) normalized to the values of the isometric reference contraction at the corresponding thumb angle for protocol 1. Force depression without prior shortening was 20.5%, whereas FD at steady‐state was significantly lower for SSC_0s and SSC_0.5s (13.7% and 15.5%, respectively). However, when a 1s interval was given between stretching and shortening, steady‐state forces (17.6%) was similar to pure FD without prior stretching. (*compared to FD; †compared to SSC_1s; p<0.05).

The steady‐state isometric force was significantly (*P* ˂ 0.05) depressed by 20.6 ± 6.6% following active shortening (FD) compared to the isometric reference contraction at the corresponding thumb angle. For the SSCs, the steady‐state isometric force was also significantly (*P* ˂ 0.05) reduced compared to the isometric reference contractions at the corresponding thumb angle. Furthermore, FD was significantly smaller (*P* ˂ 0.05) for SSC_0s and SSC_0.5s compared to the pure FD condition (13.7 ± 7.4% and 15.5 ± 2.9%, respectively), while the corresponding FD for SSC_1s (17.6 ± 6.1% force depression) was similar to the pure shortening FD contractions (Fig 3).

### Protocol 2

The mean isometric reference force at a 0° thumb adduction angle was 37.5 ± 9.3 N, representing 56% of the MVC (66.7 ± 11 N). There was a 6% loss in isometric force from before to after the SSC contractions.

As expected, owing to differences in starting lengths, there was a significant higher value of force before stretching (b‐STR) for SSC contractions compared to the initial force before shortening for pure FD contraction (*P* ˂ 0.05; Fig 2), but no difference in force among SSC contractions (*P *>* *0.05). Forces at the end of stretch (e‐STR) were similar for all SSC contraction (*P *>* *0.05). Forces at the end of shortening (e‐SHO) were significantly greater for SSC_15°/s, SSC_20°/s, and SSC_30°/s compared to the pure FD tests (*P* ˂ 0.05), but were not different (*P* > 0.05) between SSC_60°/s and the FD tests. Finally, there was significantly more work performed for all SSC compared to the FD contractions (*P* ˂ 0.05), with the highest work performed for tests with the slowest shortening speed (SSC_15°/s; Table 2).

**Figure 4:**
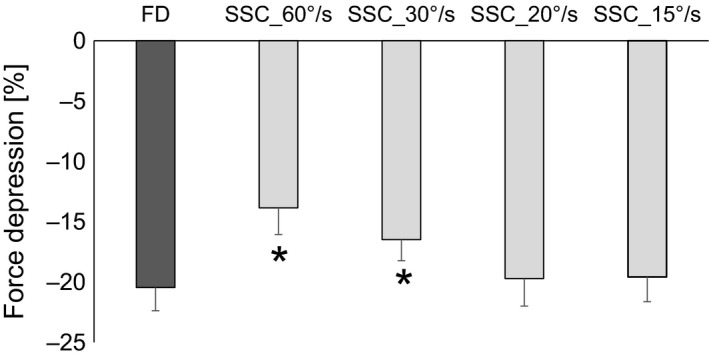
Mean (±SE) values of force decrease for pure force depression (FD) and stretching‐shortening cycles (SSC) with varying shortening speeds of 60, 30, 20, and 15°/s (SSC_60°/s, SSC_30°/s, SSC_20°/s, SSC_15°/s, respectively) normalized to the values of the isometric reference contraction at the corresponding thumb angle for protocol 2. Force depression without prior shortening was 20.3%, whereas FD at steady‐state was significantly lower for SSC_60°/s and SSC_30°/s (13.2% and 16.8%, respectively). However, at slower shortening speeds the steady‐state forces (19.6 and 20.3% for SSC_20°/s and SSC_15°/s, respectively) was similar to pure FD) without prior stretching. (*compared to FD, SSC_20°/s and SSC_15°/s; p<0.05).

The steady‐state isometric force was significantly (*P* < 0.05) depressed by 20.3 ± 1.4% following active shortening (FD) compared to the isometric reference contraction at the corresponding thumb angle. Similarly, the steady‐state isometric forces for all SSCs were significantly (*P* ˂ 0.05) reduced compared to the isometric reference contraction at the corresponding thumb angle. Additionally, FD in SSC_60°/s and SSC_30°/s was significantly reduced compared to the pure FD condition (13.2 ± 1.3% and 16.8 ± 1.2%, respectively; *P* ˂ 0.05), while FD for SSC_20°/s and SSC_15°s (19.6 ± 2.1% and 20.3 ± 1.5%, respectively) were similar to the pure FD condition (*P *>* *0.05; Fig. 4).

## Discussion

The present study was designed to investigate the time and speed dependence of shortening‐induced FD following an active stretch. We found that stretching prior to shortening affects FD in a time‐ and speed‐dependent manner, independent of the amount of work performed during shortening. Stretch followed by either immediate shortening (0 sec interval; protocol 1) or fast shortening speeds (30 and 60°/sec; protocol 2) resulted in a smaller FD compared to pure FD tests (Figs. 3, 4, respectively). However, if there is either sufficient time between stretch and shortening (1 sec interval; protocol 1) or slow shortening speeds (15°/sec and 20°/sec; protocol 2), then stretching has no attenuating effect on the subsequent shortening‐induced FD.

Previously it was reported that FD following shortening is unaffected by previous stretching of the muscle (Herzog and Leonard 2000; Lee et al. 2001). The authors could not explain this result within the boundaries of the rheological muscle model proposed by Forcinito et al. (1998), which accounts for the history‐dependent muscle properties through the engagement of a passive structural element upon activation. Similarly, models that take into account residual force enhancement following stretching and FD following shortening (Wu and Herzog 1999) predict that the force enhancement following stretching tends to offset the FD following shortening. The findings from studies that model these history‐dependent properties theoretically are in contrast to experimental results indicating that shortening‐induced FD is not affected by prior stretch (Herzog and Leonard 2000). However, our results confirm and extend those presented by Seiberl et al. (2015), providing evidence that stretch prior to shortening affects the amount of FD, at least if the time between the end of stretching and the end of shortening does not exceed about 1 sec.

#### Transient force values

The force values before stretching (b‐STR), at the end of stretching (e‐STR), and at the end of shortening (e‐SHO) were assessed in order to ensure a consistent force output throughout the testing protocol. As one would expect, force before stretching (b‐STR) was similar across all SSC in protocols 1 and 2. However, forces before stretching (b‐STR) for SSC contractions were significantly higher when compared to the force before shortening for pure FD in protocols 1 and 2, which is expected based on the force–length relationship of the adductor pollicis muscle (Jones et al. 2016). Given the stretch magnitude and stretch speed were the same for all SSC conditions (30° and 30°/sec, respectively), forces at the end of stretching (e‐STR) were similar across all SSC contractions in both protocols. Forces at the end of shortening (e‐SHO) were significantly greater for all SSC contractions compared to the pure FD tests in protocol 1. Additionally, forces at the end of shortening (e‐SHO) for SSC_1s were significantly smaller compared to those obtained in the SSC_0s tests (protocol 1). The lower forces at the end of shortening (e‐SHO) for SSC_1s might be attributed to the lower force prior to shortening compared to the SSC_0s tests. Similarly, the force at the end of shortening (e‐SHO) in protocol 2 was significantly greater for all SSC compared to the pure FD contractions, except for the fastest shortening speed condition (SSC_60°/sec). The highest shortening velocity (60°/sec) might have caused a significant drop in force due to the force–velocity relationship, reaching similar values of at the end of shortening as the pure FD tests performed with a shortening speed of 30°/sec.

#### Average work values

In protocol 1, the average work performed was significantly greater for all SSC compared to the pure FD contractions, possibly because of the increased force at the start of shortening for the SSC tests. Furthermore, the average work for SSC_0s and SSC_0.5s was significantly higher compared to the SSC_1s tests. Increasing the interval between stretch and shortening is associated with a reduction in the isometric force prior to shortening, which may contribute to the reduction in mechanical work. Similarly, average work values in protocol 2 were significantly higher for all SSCs compared to the pure FD trials. Additionally, work was greater for SSC_15°/s compared to SSC_60°/s shortening contractions. Increasing the speed of shortening is associated with reduction in work in accordance with the force–velocity relationship (Hill 1938) of muscle contraction.

### Time‐dependent FD

It has been shown previously in the cat soleus muscle that stretching prior to shortening does not affect the magnitude of shortening‐induced FD (Lee et al. 2001). In contrast, we found here that stretching prior to shortening affects FD in a time‐dependent manner. When the time between the end of stretch and the end of shortening in SSC trials is short (about 0–1.5 sec), FD is significantly reduced compared to a pure FD test. However, when the time between the end of stretch and the end of shortening is long (about 1.5 sec and more), FD is not affected by the stretch preceding the shortening. Therefore, whatever the mechanism for FD, it seems to be perturbed immediately following a stretch causing a reduced FD, but FD is fully re‐established given sufficient time between the end of the active stretch and the end of shortening.

### Speed‐dependent FD

The shortening speed in protocol 2 was adjusted in order to increase the time between the end of stretch and the end of shortening to match the times in protocol 1, without introducing a pause between the stretch and shortening segments of the SSCs. In protocol 1, the time interval between the end of stretch and end of shortening was 1, 1.5, and 2 sec for SSC_0s, SSC_0.5s, and SSC_1s, respectively. In protocol 2, the corresponding time interval was 2, 1.5, 1, and 0.5 sec for SSC performed at 15°/s, SSC_20°s, SSC_30°s, and SSC_60°/s, respectively. For the fast shortening speeds (30 and 60°/sec), the SSCs steady‐state isometric force was significantly greater compared to the force in the pure FD trials, resulting in less FD. This result agrees with the findings from protocol 1 with no time interval between SSC contractions (SSC_0s). Furthermore, slow shortening speeds (15 and 20°/sec) following stretch resulted in similar FD as the pure FD trials and similar FD as observed in the SSC_1s trials. Therefore, our results lead to the suggestion that FD following stretch–shortening cycles is affected by the time between the end of stretch and the end of shortening.

### Potential contributing mechanisms to the time and speed dependence of FD

Force depression following pure shortening contractions is predicted well by the amount of mechanical work performed by the muscle in the shortening phase (De Ruiter et al. 1998; Herzog and Leonard 2000). Previous studies have shown consistently that the greater the work performed during shortening, the greater the amount of FD (Seiberl et al. 2015; Fortuna et al. 2016). This association has been explained mechanistically with the idea that actin filaments entering the A‐band area are distorted and this distortion leads to a change in cross‐bridge attachment kinetics, thus reducing the proportion of attached cross‐bridges. This so‐called cross‐bridge inhibition theory purports that an increase in stress on the actin filaments (with increasing muscle force) and an increase in the newly formed overlap zones (with increasing shortening magnitudes) reduces the probability of cross‐bridge attachment, thus decreasing the proportion of cross‐bridges with increasing mechanical work. In the current study, we observed that all SSC experiments were associated with a greater amount of work compared to pure FD trials. Additionally, there was more work performed when no pause was given between the stretch and shortening phase of the SSC trials compared to the 1 sec pause in protocol 1, therefore one would expect more FD in the former compared to the latter. However, the opposite results were observed (Fig 3). Similarly, in protocol 2, decreasing the shortening speed, thus increasing the time between stretch and shortening produced similar results as observed in protocol 1. We speculate that the reason why we found these contrasting results with the literature is based on two competing mechanisms for residual force enhancement and FD: (1) force enhancement due to stretch before shortening and (2) work performed during shortening. The results from this study suggest that force enhancement due to stretch (and the effects of this enhanced force state on FD) decreases as a function of time. In other words, increasing the interval between stretch and shortening can reduce the effects of force enhancement on the steady‐state isometric force following shortening, and if given sufficient time, the enhancement effect is completely abolished.

## Conclusion

In the present study we show that stretch preceding shortening affects FD in a time‐ and speed‐dependent manner. The results reported here provide novel information on the potential mechanism behind the history‐dependent properties of force enhancement and FD, as the events occurring during active muscle stretching either inhibit full development of FD or offset FD in some hitherto unknown manner.

## Conflict of Interest

The authors have no conflicts of interest to disclose.
